# Cytochrome P450 aromatase expression in human seminoma

**DOI:** 10.1186/1477-7827-3-72

**Published:** 2005-12-22

**Authors:** Vittoria Rago, Francesco Romeo, Saveria Aquila, Daniela Montanaro, Sebastiano Andò, Amalia Carpino

**Affiliations:** 1 Cell Biology Department, Faculty of Pharmacy, University of Calabria, Italy; 2 Pathologic Anatomy Unit, Annunziata Hospital, Cosenza, Italy; 3 Pharmaco-Biology Departments, Faculty of Pharmacy, University of Calabria, Italy

## Abstract

**Background:**

The enzyme cytochrome P450 aromatase, catalysing the conversion of androgens into estrogens, has been detected in normal human testicular cells suggesting a physiological role of local estrogen biosynthesis on spermatogenesis control. Estrogens, regulating cell growth and apoptosis, can also be involved in tumorigenesis process, but the possible link between estrogens and testicular neoplastic process is, up to now, scarcely known. This study examined aromatase expression in human seminoma, which is the most common germ cell tumour of the testis.

**Methods:**

The tumour-bearing testes were obtained from 20 patients with classic seminoma undergoing to therapeutic orchidectomy. Paraffin embedded tissues were processed for immunohistochemistry using a mouse monoclonal antibody generated against human placental cytochrome P450 arom, as primary antibody, and a biotinylated goat-anti-mouse IgG, as secondary antibody. Furthermore, Western blot analysis of seminoma extracts was carried out.

**Results:**

Intense P450 arom immunoreactivity was observed in the seminoma cells and Western blot analysis confirmed the immunodetection. A strong immunostaining was also detected in cells of intratubular germ cell neoplasia (IGCN), adjacent to seminoma.

**Conclusion:**

The present study demonstrated, for the first time in human, aromatase expression in neoplastic cells of seminoma suggesting a relation between local estrogen biosynthesis and germ cell tumorigenesis. The P450 arom immunolocalization in the cells of IGCN, representing the common precursor of most germ cell tumors, seems to support these findings.

## Background

Somatic and germ cell tumors are typical testicular neoplasms which can affect young, adult and elderly men. In the last years, testis tumor management has improved by new diagnostic and surgical tools, as well as by innovative therapy options to preserve fertility against cytotoxic treatments [1]. However, pathogenesis of male gonad malignancies is often still unknown.

Somatic and germ cells of normal human testis have revealed the expression of estrogens receptor-β and /or aromatase, which is the microsomial enzyme catalysing the conversion of androgens into estrogens [2-8]. Therefore, testicular cells are considered targets and in situ biosynthesis sites of estrogens, suggesting a physiological role of these hormones in steroidogenesis control and sperm maturation.

Estrogens are also known to regulate cell growth and apoptosis, therefore they can be involved in tumorigenesis process [9,10] but, up to now, their possible link with testicular neoplastic process is scarcely known.

Germ cell tumors, comprising seminoma and non-seminoma, are the most common malignancies among human male aged 15–40 years. Aim of this work was to investigate aromatase expression in seminoma which represents approximatively the 40% of these testicular neoplasms.

## Methods

### Samples and histopathological studies

The archival cases (collected during the last 3 years) were provided by the Pathologic Anatomy Unit (Annunziata Hospital). Tumoral testicular tissues were obtained from 20 patients (ages from 27 to 38 years) with unilateral classic seminoma undergoing to therapeutic orchidectomy. Normal testicular tissues were obtained from 2 patients showing testes with a granulomatous lesion. The ethical committee members of the University of Calabria approved the investigation programme.

Tissues were fixed in formalin (4%), dehydrated with a series of ethanol solutions and paraffin-embedded. Paraffin sections, 5 μm thick, were mounted on slides precoated with polylysine, deparaffinized and rehydrated (7–8 serial sections for each sample). These sections were used for morphological and immunohistochemical analyses.

Histopathological studies were carried out by Haematoxylin-Eosin staining. Seminoma samples were also investigated by placental-like alkaline phosphatase (PLAP) immunohistochemistry (monoclonal mouse anti human PLAP, clone 8A9, DAKO-Cytomation, Milan, Italy)

Furthermore, 50 μm thick [11] paraffin serial sections of seminoma specimens were cut for protein extraction and mounted on glass slides.

### P450 arom immunohistochemistry

Immunohistochemistry was performed after heat-mediated antigen retrieval [12]. Hydrogen peroxide (3% in distillate water) was used, for 30 minutes, to inhibit endogenous peroxidase activity while normal goat serum (10%) was utilised, for 30 minutes, to block the non-specific binding sites. P450 arom immunodetection was carried out using a monoclonal mouse anti-human cytochrome P450 aromatase (MCA 2077, Serotec, Oxford,UK) (1:50 in TBS) at 4°C overnight. Then, a biotinylated horse-anti-mouse IgG (Vector Laboratories, CA, USA) was applied (1:600 in TBS) for 1 hour at RT, followed by the avidin-biotin-horseradish peroxidase complex (ABC/HRP) (Vector, Laboratories, CA, USA). Immunoreactivity was visualized by using the diaminobenzidine chromogen (DAB) (Zymed Laboratories, CA, USA) and, finally, sections were counterstained with haematoxylin. The primary antibody was replaced by normal rabbit serum in negative control, while the absorption control has utilised a primary antibody preabsorbed with an excess (5 nmol/ml) of purified human placental aromatase protein (4°C for 48 hours). Rat ovary sections were used as positive control. 6–7 serial sections were processed for each sample.

### Protein extraction

Protein extraction from formalin-fixed paraffin-embedded sections has been carried out according to Ikeda [11]. Briefly, 50 μm testis sections from seminoma regions were deparaffinized in xylene, reydrated in graded ethanol, immersed in distilled water, and air dried. Then, the neoplastic area was recovered from the glass slides, further it was cut into small pieces and placed in Eppendorf tubes. Two hundred μl of RIPA buffer, pH 7,6 (1 M NaH_2_PO_4_, 10 mM Na_2_HPO_4_,154 mM NaCl, 1% Triton X-100, 12 mM C_24_H_39_O_4_Na, 0.2% NaN_3_, 0.95 mM NaF, 2 mM PMSF, 50 mg/ml aprotinin, 50 mM leupeptin) (Sigma Chemical, Saint Louis, MO,USA) containing 0.2% SDS, was added to each tube and the contents were incubated at 100°C for 20 minutes, followed by incubation at 60°C for 2 hours. After incubation, tissue lysates were centrifuged at 15.000 × g for 20 minutes at 4°C and the supernatants were stored at -80°C until biochemical analysis.

### Western blot analysis

Tissue lysates were quantified using Bradford protein assay reagent [13]. Equal amounts of protein (50 μg) were boiled for 5 minutes, separated under denaturing conditions by SDS-PAGE on 10% polyacrylamide Tris-glycine gels and electroblotted to nitrocellulose membrane. Non-specific sites were blocked with 5% non fat dry milk in 0.2% Tween-20 in Tris-buffered saline (TBS-T) for 1 hour at room temperature and then probed with a rabbit polyclonal antiserum generated against human P450 aromatase(Hauptman-Woodward Medical Research Institute, Buffalo, NY, USA) (1:750). After extensive washings (three times for 15 minutes each time in TBS-T), a goat-antirabbit (1:7000) horseradish peroxidase-conjugated antibody (Vector Laboratories, CA, USA) was added for 1 hour at 22°C. Blots were again washed three times for 15 minutes in TBS-T and the bound of secondary antibody was located with the ECL Plus Western blotting detection system according to the manufacturer's instructions. Each membrane was exposed to the film for 2 minutes. P450 arom protein, isolated from human placenta, was used as positive control. Negative control was prepared using tissue lysate, where P450 arom was previously removed by preincubation with P450 arom antibody (1 hour at room temperature) and subsequently immunoprecipitated with protein A/G – agarose.

## Results

### Histopathological study

**Figure 1:**
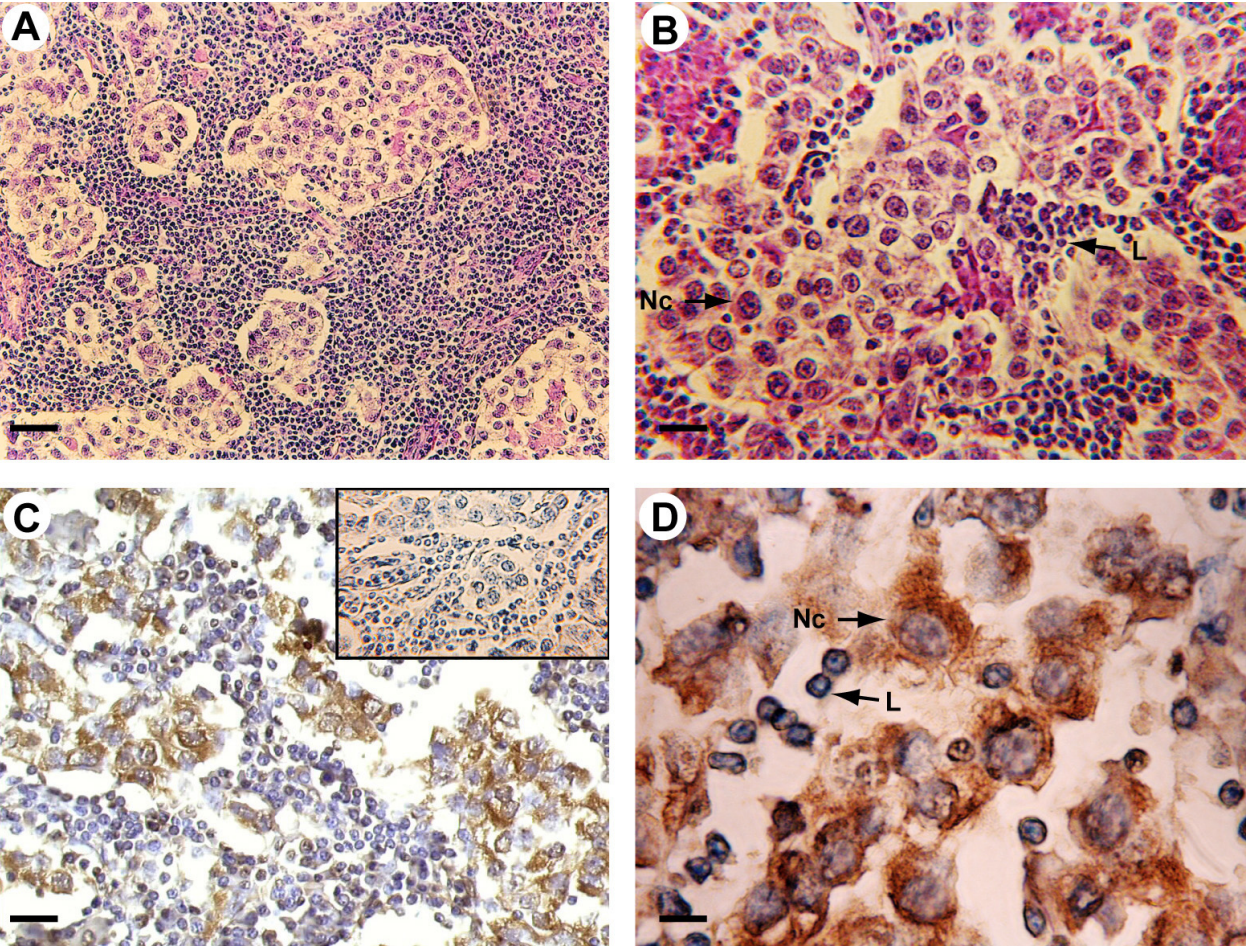
Morphology and P450 arom immunoreactivity of tumoral region in human testis with seminoma. A-B: Haematoxylin-eosin staining. C-D: Strong P450 arom immunoreactivity in cytoplasm of neoplastic cells (Nc) and unstained lymphocytes (L). Insert: absorption control. Scale bars: A, 20 μm; B-C, 12.5 μm; D, 5 μm.

Tumoral regions of all specimens have revealed the presence of classic seminoma with a uniform population of large neoplastic cells and extensive leukocytic infiltration (Fig 1A,B). All seminoma specimens showed tumor stage I.

The region adjacent to seminoma (60% of samples) has shown the presence of abnormal seminiferous tubules with decreased tubular diameters and lacking spermatogenesis, which were identified as intratubular germ cell neoplasia (IGCN). These intratubular lesions were characterised by the basal proliferation of undifferentiated atypical enlarged germ cells, with big nuclei, and by the presence of Sertoli cells in luminal displacement.

**Figure 2:**
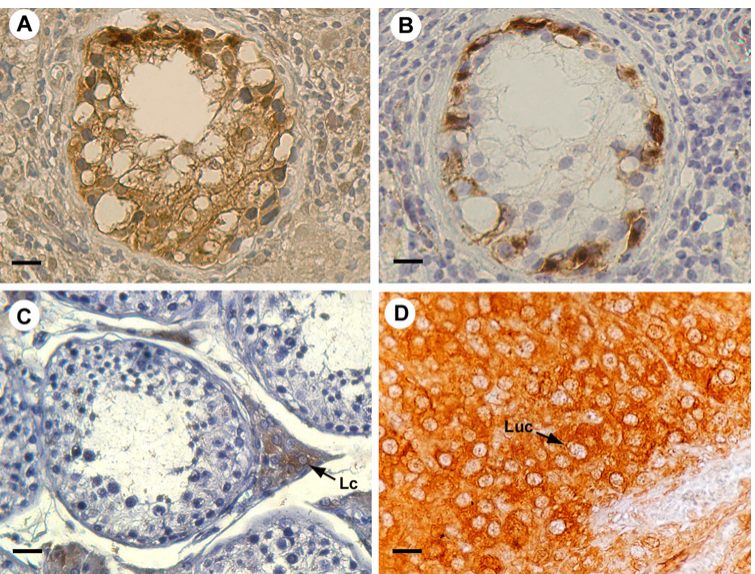
P450 arom immunoreactivity in testicular region adjacent to seminoma and in controls A: Intense aromatase immunostaining in IGCN cells. B: Placental-like alkaline phosphatase staining of IGCN basal cells C: Strong aromatase immunoreactivity of interstitial Leydig cells in normal testis (Lc) D: Intense immunostaining of luteal cells (Luc) in ovarian tissue Scale bars: A-B, 8 μm; C,12.5 μm; D, 5 μm.

The IGCN diagnosis was confirmed by PLAP immunohistochemistry. In fact, cytoplasmic and membranous dark staining have revealed the PLAP immunopositivity in the malignant cells aligned along the basal portion of seminiferous tubules (Fig 2B), indicating their germ cell origin.

Furthermore, normal testes have displayed typical normal seminiferous tubules, with active spermatogenesis.

### P450 arom immunolocalization

P450 arom immunoreactivity in testicular cells was detected as cytoplasm staining while the nuclei were immunonegative.

In tumoral regions, neoplastic cell cords have revealed a strong immunoreactivity, while the surrounding abundant lymphocytes and connective cells were all immunonegative. The immunostaining pattern was similar in all the 20 examined samples and pictures in the figures show representative specimens (Fig 1C,D). In all the 12 testis samples with intratubular germ cell neoplasia, an intense immunostaining has been observed in the cells of IGCN, adjacent to seminoma, either in the abnormal germ cells or in Sertoli cells (Fig 2A). Leydig cells, clustered in interstitial tissue among IGCN, were strongly immunoreactive, representing an internal positive control (data not shown). Furthermore, the 2 normal testes have revealed an intense immunoreactivity in Leydig cells, while tubular compartments were all completely immunonegative (Fig 2C).

Negative (data not shown) and absorption controls (insert of figure 1C) were all immunonegative. As expected, ovarian tissue has shown a strong immunoreactivity in luteal cells (positive control) (Fig 2D).

### Western blot analysis

**Figure 3:**
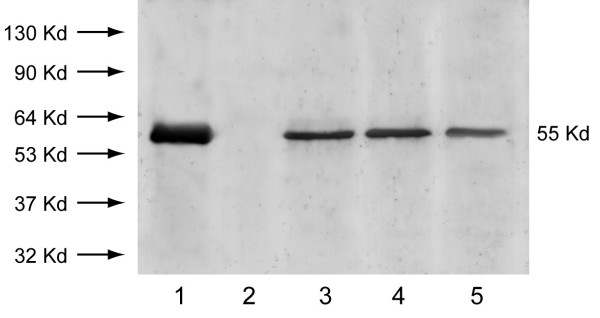
Immunoblot of aromatase from seminoma extracts. *Lane *1: purified P450 arom protein from human placenta (positive control). *Lane *2: negative control prepared as described in Material and Methods. *Lanes *3, 4, 5: 55 kDa P450 arom immunoreactive bands from lysates of three different seminoma extracts. *Numbers *on the left correspond to molecular weights of marker proteins.

Lysates of all the 20 formalin-fixed paraffin-embedded testis sections from seminoma tumoral regions have been submitted to Western blot analysis. All specimens have shown a single band corresponding to a molecular mass of 55 kDa, which is the correct size of human P450 arom protein (3 representative samples are shown in Fig 3). The band migrated at the same mobility as purified human placental P450 arom protein which was used as positive control, while the immunoreactive P450 arom band was lacking in the negative control (Fig 3)

## Discussion

The present study has demonstrated, for the first time, aromatase expression in neoplastic cells of human seminoma, which is the most common testicular germ cell tumor of men in reproductive age.

Aromatase detection in Leydig cells, Sertoli cells and elongated spermatids of normal human testis has suggested the involvement of local estrogen production in gonadal physiology [3,4,7]. At the same time, the mitogenic estrogen action could be related to the testicular tumor etiology, as hypothesized for human breast, ovarian, endometrial and epathic malignancies [14,15].

Epidemiological studies have indicated a higher incidence of testicular cancers after prenatal estrogen exposure [16-19]. Furthermore, a link between exposure to estrogens/estrogen mimics and testicular cancer risk has been hypothesized [20,21].

Few studies have investigated aromatase in testicular tumors. Among somatic testis tumors, a large cell calcifying Sertoli cell tumor [22] and Sertoli cell tumors of Peutz-Jeghers syndrome have shown an enhanced expression of this enzyme [23,24]. Furthermore, the single Nakazumi's report on testicular germ cell tumors has not detected aromatase expression in neoplastic cells of seminomas and non-seminomas but it revealed the enzyme presence only in stromal cells of non seminomas [25].

The present study has shown aromatase immunolocalization in seminoma cells and Western blot analysis has confirmed this result. Our data disagree with Nakazumi's reports, but probably, the recent antibodies, used in our investigation, have improved aromatase immunodetection. In addition, our findings agree to a very recent work demonstrating the aromatase expression in human testicular seminoma cell line, JKT-1 [26].

Furthermore, a strong P450 arom immunoreactivity has been observed inside the abnormal tubules of IGCN, which represents the common precursor of most germ cell tumours. Invasion of the tubular wall by malignant germ cells or intratubular proliferation of tumour cells are the two theories proposed to understand the switch from IGCN to testicular neoplasia [27,28]. However, to date, the mechanisms of invasive tumour development from pre- invasive lesion are still to be elucidated. P450 arom presence in malignant germ cells and in Sertoli cells of IGCN is a new finding which supports aromatase expression during seminoma development and suggests a possible role of local estrogen biosynthesis in tumorigenesis process. The aromatase absence in germ and Sertoli cells inside seminiferous tubules of normal testes supports this hypothesis. Concerning normal testes, aromatase detection in Leydig cells agrees with previous reports [3,7], but its absence in seminiferous tubule cells could disagree with Turner's paper [7] indicating a "possible" P450 arom expression in elongated spermatids and a "faint immunopositive reaction in Sertoli cells". However, the same authors claimed the immunostaining "less convincingly specific in human" than in marmoset and rat.

## Conclusion

Finally, the present study is the first report of aromatase expression in human testicular germ cells after malignant transformation, indicating that estrogens, from in situ aromatization, could act as autocrine mitogenic factors promoting neoplastic growth. These findings could represent a cue for further studies on estrogen involvement in testicular germ cell tumorigenesis and new pharmacological treatment.

This work was supported by "Ministero dell'Universitàe della Ricerca Scientifica e Tecnologica" (Murst 60%) and by PRIN 2004 prot.n^0^0067227.

## Authors' contributions

**CA: **the author responsible for conception, design, analysis and interpretation of data as well as of drafting manuscript.

**RV: **the author responsible for performing the immunohistochemical expriments and participating in the analysis and interpretation of data.

**RF: **the author responsible for histoplathological diagnosis and sample collection

**AS **and **MD: **the authors responsible for performing Western blot analysis and protein extraction as well as for participating to the interpretation of data.

**ANS: **the author responsible for a critical revision of the manuscript.
